# Are Pre and Postoperative Platelet to Lymphocyte Ratio and Neutrophil to Lymphocyte Ratio Associated with Early Postoperative AKI Following CABG?

**DOI:** 10.21470/1678-9741-2019-0482

**Published:** 2020

**Authors:** Mesut Engin

**Affiliations:** 1 Department of Cardiovascular Surgery, University of Health Sciences, Mehmet Akif İnan Training and Research Hospital, Karaköprü/Şanlıurfa, Turkey. E-mail: mesut_kvc_cor@hotmail.com

Dear Editor,

I have read with much interest the article of Parlar et al.^[1]^ entitled “Are Pre and Postoperative Platelet to Lymphocyte Ratio and Neutrophil to Lymphocyte Ratio Associated with Early Postoperative AKI Following CABG?”. First of all, I congratulate the authors for their invaluable contribution to the literature.

Neutrophil lymphocyte ratio (NLR) and platelet lymphocyte ratio (PLR) have been investigated in the pathogenesis and progression of cardiovascular diseases, and many studies have found valuable results in the literature. After cardiopulmonary bypass, the complement system is activated, and as a result, neutrophil counts increase, but lymphocyte and platelet counts decrease. In addition, I think that platelet values should be evaluated differently, especially after a major surgery, such as cardiac surgery. According to a study on 4,217 patients who underwent coronary artery bypass surgery, low platelet counts in the postoperative period was found to be associated with early mortality^[2]^. Also, platelet depletion is known as an indicator of the severity of critical illness^[3]^. And, in cardiac surgery and other major surgeries, the decrease in platelet counts, together with the increase in NLR, has been shown to be effective on early mortality and renal failure^[4,5]^.

The authors evaluated blood parameters in the preoperative and postoperative 1^st^, 3^rd^, and 7^th^ days. As mentioned in the last paragraphs of the discussion section, the studies on PLR are mostly presurgical/preoperative evaluations. There is no literature on PLR about the early postoperative effects after major surgery. Although the rate of PLR is found to be increased due to low lymphocyte levels in the postoperative period, it may be misleading to use this rate in the early postoperative period. These valuable findings of the authors will shed light on new studies. However, the poor prognostic significance of platelet deficiency after cardiac surgery may be more valuable, it has been also detected in other major surgeries^[5]^.
